# Association between Physical Activity and Neighborhood Environment among Middle-Aged Adults in Shanghai

**DOI:** 10.1155/2013/239595

**Published:** 2013-04-17

**Authors:** Rena Zhou, Yang Li, Masahiro Umezaki, Yongming Ding, Hongwei Jiang, Alexis Comber, Hua Fu

**Affiliations:** ^1^MOE Key Lab for Public Health and Safety, School of Public Health, Fudan University, Shanghai 200032, China; ^2^Department of Human Ecology, University of Tokyo, Tokyo 113-0033, Japan; ^3^Research Institute of Humanity and Nature, Kyoto 603-8047, Japan; ^4^Department of Geography, University of Leicester, Leicester LE1 7RH, UK

## Abstract

*Objective*. To determine the perceived neighborhood environment (NE) variables that are associated with physical activity (PA) in urban areas in China. *Methods*. Parents of students at two junior high schools in Shanghai, one downtown and the other in the suburbs, were recruited to participate in the study. They completed an International Physical Activity Questionnaire (IPAQ) and Neighborhood Environment Walkability Scale-Abbreviated (NEWS-A) survey. Participant physical activity was also objectively measured using accelerometers. *Results*. Participants from downtown areas were more positively associated with transportation PA and leisure-time PA than respondents living in the suburbs. Residential density was found to be a significant positive predictor of recreational or leisure-based PA. Street connectivity was negatively associated with leisure time PA for respondents. Moderate-vigorous PA was found to be negatively associated with traffic safety. There were no significant associations between environmental factors and transportation PA. Women had higher levels of moderate-vigorous PA than men. *Conclusions*. The results of this study demonstrate that residential density, street connectivity, and traffic safety have a significant impact on Chinese middle-aged adults' PA, suggesting urban planning strategies for promoting positive public health outcomes.

## 1. Introduction

Much research has identified and quantified the health benefits of physical activity (PA) [1]. For example, increased PA has been found to reduce the risk of cardiovascular diseases, diabetes, cancers, osteoporosis, and depression [2]. One interesting trend found in countries with emergent economies is a consistent decline of PA associated with economic development [3]. China is experiencing a process of transition from a developing country to a developed one, with increases in urbanization/urban living and associated increases of physical inactivity [4, 5]. For example, average weekly physical activity among adults in China fell by 32% between 1991 and 2006 [5] and car ownership increased from 0.5% to 13.1% between 2000 and 2010 [6]. Some variation with age has been identified: participation in exercise by all Chinese residents was 14.1% but was the lowest amongst the 18–44 age group at 5.9%. The U-shape trend of PA with age is also reflected locally and data from 2005 [7] showed that participation in exercise by Shanghai residents was the least at around 40 years of age. These data and trends indicate an urgent need to consider factors influencing middle-age adults' PA in China. 

Many studies have considered the impact on PA of the local environment in terms of its residential density, land use configuration, street connectivity, walking or cycling facilities, and aesthetics [8–11]. A number of interesting associations specifically have been identified including the relationships between residential density with walking behaviors [12, 13], increased transportation PA in areas of greater land use diversity [14], and access to recreational facilities positively associated with leisure time PA [15]. A number of literature reviews have summarized the evidence from many studies that have been published in this area [16, 17]. 

Some previous studies in China have examined individual behavior in relation to PA [18], but only a few examples have considered how the environment affects PA [19, 20]. However, the application of standard (Western) methodologies in China may not be appropriate due to both cultural differences and differences in the built environment. Some studies have examined subjective perceptions of PA with measured PA behaviors and how they relate to environmental factors and typical analyses assess transportation PA, leisure time PA, or moderate-vigorous physical activity (MVPA) [21–24]. 

In this study, subjective and objective methods were used to investigate the association between PA and neighborhood environment (NE) among middle-aged adults in Shanghai and to analyze the relative contributions of environmental variables in explaining PA in different NE contexts. The need for such research relates to urbanization in China now exceeding 50% to provide evidence relating to NE impacts on PA and inform urban policy making in China.

## 2. Methods 

### 2.1. Study Design

Research was conducted as part of a cross-sectional study to investigate the spatial patterns of PA in Shanghai (the Evaluation of Spatial-Patterns of Physical Activity—ESPA project). Two typical junior high schools in Shanghai were selected as case studies: one in the downtown area (Changning district) and another in the suburbs (Pudong district). The parents of the grade-2 students in junior high school were recruited as subjects. Data were collected between October 2010 and June 2011. The study was approved by Ethics Committee of Fudan University, and the University of Tokyo and the participants had given written informed consent. 

### 2.2. Study Procedure

Parents were asked to fill a structured questionnaire of their individual characteristics (age, gender, weight, height, educational background, family income), a Chinese version of the NEWS-A (Neighborhood Environment Walkability Scale-Abbreviated) survey and the IPAQ (International Physical Activity Questionnaire-Long) survey. 

With the purpose of finding the household effect, plus the lack of the accelerometer devices, adults in two-parent families were selected to wear the accelerometer (Lifecorder EX (Suzuken Co., Ltd, Nagoya, Japan)) in order to evaluate their physical activity levels. However, there were still a few adults in one-parent families who were included into the accelerometer survey due to the operational reasons. Participants were instructed to attach the accelerometer to an adjustable belt and to wear it firmly around their waist, positioned just above the right hip. Participants were instructed to wear the accelerometer consecutively for 7 days except when sleeping or engaged in water-related activities such as swimming and showering. Class teachers reminded the participants of the correct way to wear the accelerometer and encouraged them to continue to wear it for the direction of the study.

### 2.3. Measures

The NEWS-A questionnaire captures measures of respondent perceptions of their neighborhood environment using a four-point scale. From this data standard measures are generated to describe residential density, the diversity of land use, facility access, street connectivity, walking and cycling facilities, the aesthetics of the environment, pedestrian safety, and crime safety [25]. IPAQ-long form is designed to measure cross-national PA in adults and considers work-related PA, transport PA, domestic and gardening PA, and leisure PA. It asks respondents to describe their time spent on walking, moderate PA and vigorous PA within each domain. The reliability and validity of the Chinese version of NEWS-A and IPAQ have been demonstrated in previous studies [25, 26].

Objective PA data for individuals can be obtained using an accelerometer. This study used the Lifecorder EXwhich has been shown to measure PA and energy expenditure at a range of different activity levels [27, 28]. 

In order to obtain valid PA measurements [29], wear and nonwear times were defined as follows: nonwear time as a period of at least 60 consecutive minutes of zero PA level; any period of less than 60 consecutive minutes not recorded as nonwear time. Wear time as a minimum period of 10 hours (8:00–18:00) with an absence of nonwear time during that period was defined as a valid day. Data for participants with at least 1 valid day were included in the analysis.

In order to ensure data quality and completeness, questionnaires with missing data were returned to the participants and any questionnaires that were still incomplete after this were excluded from the final analysis. Double data entry was used. Epidata 3.1 was used to enter the questionnaire data and Predictive Analytics Software (PASW) 18.0 was used for data editing and analysis. 

### 2.4. Analysis

The analysis sought to investigate the relationships between perceived transportation and leisure time physical activity, as captured by the IPAQ survey, and environmental and demographic variables. The data on self-reported transportation PA (i.e., physical activity related to “getting somewhere”) and self-reported leisure time PA (i.e., recreational physical activity) were modeled against NEWS-A attributes such as residential density, land use diversity, facility access, street connectivity, walking and cycling facilities, the aesthetics of the environment, pedestrian traffic safety, and crime safety (Model A), and then this analysis was extended by considering participant demographics (Model B). Work-related PA and domestic and gardening PA were not included in this analysis because these two domains of PA were affected by lots of social and individual factors, and there were few studies showing their significant association with neighborhood environment. Actual physical activity data as captured by the accelerometer was also compared with environmental variables (Model A) and environmental variables plus demographic attributes (Model B). 

Unadjusted and multivariate-adjusted odds ratios (OR) and 95% confidence intervals (CI) were calculated from logistic regressions to examine the association between NE and PA. Eight environmental variables were used as the independent variables to estimate dependent variables of transportation, leisure time PA, and moderate-vigorous physical activity. The models were adjusted for area, gender, educational background, and family income level. For self-reported transportation and leisure time PA, participants were divided into two groups (low active and active) depending on whether they met the recommendations of the IPAQ international criterion. For the accelerometer measured PA, MVPA was divided into two groups based around the median value for all participants: >=36 minutes/day and <36 minutes/day. 

## 3. Results

Of the 515 questionnaires sent out, 478 (92.8%) valid ones were collected and analyzed. Of these, 327 respondents from two-parent families were asked to wear the accelerometer, with 235 having valid accelerometer data.

Table 1 presents a breakdown of the demographics of the participants in the questionnaire and accelerometer-based surveys. The sample for each survey included 48% of male, mean age (SD) was 40 (6) years, and 33% of participants were overweight or obese (body mass index was greater than or equal to 24). Almost half of the questionnaire participants had a family income of more than 40,000 RMB per year (47.6%), with the national average being 42,500, and had an educational background of at least high school level (43.5%). Of the participants in the accelerometer survey, 49.8% had an income greater than 40,000 RMB per year and 44.9% had an educational background of at least high school level. 

The attributes of the respondent PA and NE are presented in Table 2. This shows that the transportation PA active group included 42.5% of the participants and that the leisure time PA active group included 25.3% of the participants. No significant differences were found between genders in the perception of transportation PA, leisure time PA, and neighborhood environment, but a difference was found in MVPA between male and female respondents with female having significantly higher levels of MVPA than male. 

Logistic regression was used to identify the factors influencing PA (Table 3). For Model A, respondents' transportation PA was not found to be associated with any of the neighborhood environmental variables. When demographic variables were considered alongside environmental ones (Model B), the associations between transportation PA and the environmental variables were still not present, but participants from the downtown area were found to be more positively associated with transportation PA than respondents living in the suburbs. 

For both the unadjusted and adjusted models, higher levels of leisure-time PA were positively associated with perceived residential density and with poorer neighborhood street connectivity. In the demographically adjusted model (Model B), participants from the downtown area were more positively associated with leisure time PA than those living in the suburbs. Residential density was a significant positive predictor, while street connectivity was a negative predictor of recreational or leisure-based physical activity in both the unadjusted and demographic adjusted models.

MVPA was found to be negatively associated with traffic safety in Model A. After adjusting for demographic variables, the results indicated that women were more strongly associated with increased levels of MVPA than men; however, there was no association between MVPA and traffic safety in the adjusted model. 

## 4. Discussion

The results indicate possible associations between traffic safety and MVPA among Chinese adults (parents) and that two neighborhood environmental variables (residential density, street connectivity) are significantly associated with leisure time PA after adjusting the demographic variables. The impacts of environmental factors vary depending on the nature of the PA. Leisure time PA was found to be positively associated with residential density but negatively associated with street connectivity, and MVPA was found to be negatively associated with traffic safety. 

Although many people (more than 20%) in China walk to work (transportation PA) compared to less than 5% in western countries [30], with the improvement of public transportation and the growth of private motor vehicles, it is likely that less people in the future will walk or cycle to work. Other studies have suggested that physical environment may have a greater effect on transportation PA than leisure time PA. Learnihan et al. [31] found land use diversity to be the most critical factor influencing transportation PA. However, land use diversity and other environmental variables were not found to be associated with transportation PA in this study. This may be due to the different effects in suburb and downtown cancelling each other out as exemplified by land use diversity which was positively associated with suburban participants' transportation PA but negatively associated with downtown participants' transportation PA. 

In urban China, the per capita living space is 31.6 m^2^, with a population density of 2209 people per km^2^, whilst in Shanghai, the population density is 3630 people per km^2^ [6]. In this study, the higher levels of residential density in the participant neighborhood environments were associated with higher levels of leisure time PA (with or without demographic adjustment). This is consistent with other studies [32] which have also found higher housing density to be positively associated with physical activity. However, a Japanese study [12] has shown that high residential density is associated with reduced leisure PA for women, indicating that the correlates may be due to gender or cultural differences. This study found that women did more MVPA than men, yet in other studies women have been found to be less active with less MVPA than men in global studies [30], suggesting that further studies may be needed to unpick these differences.

Van Dyck et al. [9] found perceived street connectivity to be negatively associated with recreational walking. This is consistent with the findings of this research, which showed poorer street connectivity to be associated with more leisure-time PA. This suggests for neighborhoods with better street connectivity that greater consideration should be given to interventions for improving the opportunities for leisure-time PA. 

The results of previous studies investigating the relationships between traffic safety and PA are not consistent. Some studies have variously shown traffic safety to not be related to PA [33], others has shown that it is positively associated [12, 34] and yet further studies have found it to be negatively associated [23]. This research found that higher perceptions of traffic safety problems were associated with less MVPA, although this association was not found when the model was adjusted for socioeconomic variables (Model B). That is, high perceptions of traffic safety issues resulted in less PA, which may be due to a dependence between perceptions of traffic safety and environmental attributes with, for example, areas with heavy traffic often having a greater diversity of land uses. 

There were differences in the associations with PA between respondents from different areas. For example, respondents from downtown areas were found to be more positively associated with transportation PA and leisure time PA than respondents living in the suburbs. It should be noted that parents of children from only one school were selected in each area and the results may be not representative, especially as some of the effect sizes are small. This may limit their practical relevance, but they do indicate possible interventions in relation to improving PA especially in relation to active commuting to work and leisure time in the suburbs. Future studies will be conducted in more schools to verify the results. 

There are several limitations in this study. First, the study was cross-sectional and the direction of causality could not be evaluated, suggesting the need for longitudinal or intervention studies in the future. Second, the environmental attribute measures were derived from subjective questionnaires rather than objective measures from a GIS or remote sensing analysis [35]. Third, the age range of the participants was narrow, which limits the extrapolation of the findings to other age groups. Fourth, the participants were selected from the parents of children at only one school in each area, which may not fully represent the population of Shanghai and may limit the generalization of the results to other areas. 

## 5. Conclusions 

This study confirms the relationship between residential density, street connectivity, and traffic safety with a range of PA behavior in middle-aged Chinese adults. No significant associations were detected between transportation PA and environmental variables. Leisure time PA was found to be positively associated with residential density but negatively with street connectivity. The perceptions of traffic safety were strongly associated with MVPA, although this association disappeared after the model was adjusted for socioeconomic variables. The results suggest a number of ways that urban planning could increase the physical activity level of the middle-aged adults in China, for example, by centralizing the residence in the neighborhood, especially in downtown areas. 

## Figures and Tables

**Table 1:** Demographics of the participants in the questionnaire-based survey and accelerometer-based survey.

	Questionnaires- based survey	Accelerometer- based survey
Participants number	478	235
Age^a^	39.8 ± 6.29	39.6 ± 5.64
Gender^b^		
Male	231 (48.3)	113 (48.1)
Female	247 (51.7)	122 (51.9)
Area^b^		
Suburb	231 (48.3)	117 (49.8)
Downtown	247 (51.7)	118 (50.2)
BMI^b^		
<18	16 (3.4)	3 (1.3)
≥18, <24	300 (63.2)	154 (66.1)
≥24, <28	127 (26.7)	61 (26.2)
28≤	32 (6.7)	15 (6.4)
Family income^b^		
<20,000 RMB	99 (20.8)	43 (18.5)
2–40000 RMB	150 (31.6)	74 (31.8)
40000 RMB<	226 (47.6)	116 (49.8)
Educational background^b^		
Primary school	18 (3.8)	8 (3.4)
Junior high school	250 (52.7)	121 (51.7)
High school	93 (19.6)	46 (19.7)
College	89 (18.8)	44 (18.8)
University and above	24 (5.1)	15 (6.4)

^a^data presented as means ± SD; ^b^data presented as *N* (%).

**Table 2:** Comparison of physical activity and neighborhood environment of participants using IPAQ, NEWS-A, and accelerometers by sex.

	Male	Female	Overall
IPAQ-based physical activity indicators^a^			
Transportation PA			
Active group	99 (42.9)	104 (42.1)	203 (42.5)
Low active group	132 (57.1)	143 (57.9)	275 (57.5)
Leisure time PA			
Active group	57 (24.7)	64 (25.9)	121 (25.3)
Low active group	174 (75.3)	183 (74.1)	357 (74.7)
Accelerometer-based physical activity indicators^a^			
MVPA (minutes per day)*			
36-	49 (43.4)	71 (58.2)	120 (51.1)
-35	64 (56.6)	51 (41.8)	115 (48.9)
Perception of neighborhood environment using the NEWS-A^b^			
Residential density	348.8 ± 130.6	352.1 ± 130.9	350.5 ± 130.6
Land use mix diversity	2.88 ± 0.81	2.85 ± 0.81	2.86 ± 0.81
Land use mix access	2.88 ± 0.60	2.88 ± 0.58	2.88 ± 0.59
Street connectivity	2.82 ± 0.53	2.80 ± 0.51	2.81 ± 0.52
Walking/cycling facilities	2.68 ± 0.72	2.65 ± 0.70	2.67 ± 0.71
Aesthetics	2.69 ± 0.78	2.70 ± 0.75	2.69 ± 0.76
Traffic safety	2.85 ± 0.52	2.86 ± 0.52	2.86 ± 0.52
Crime safety	3.05 ± 0.91	2.98 ± 0.90	3.01 ± 0.90

*P < 0.05 MVPA, moderate-vigorous physical activity; ^a^data presented as *N* (%); ^b^data presented as means ± SD.

**Table 3:** Association between physical activity and neighborhood environment measures.

Correlates of PA	Self-reported PA (IPAQ)				Accelerometer-measured PA	
	Transportation PA		Leisure time PA		MVPA	
	(low active group and active group)		(low active group and active group)		(≥36 minutes/day group and <36 minutes/day group)	
	Model A^a^ OR (95% CI)	Model B^b^ OR (95% CI)	Model A OR (95% CI)	Model B OR (95% CI)	Model A OR (95% CI)	Model B OR (95% CI)
Area						
Suburb		Reference		Reference		Reference
Downtown		2.127 (1.212, 3.734)**		2.110 (1.100, 4.048)*		1.224 (0.516, 2.900)
Sex						
Male		Reference		Reference		Reference
Female		0.960 (0.654, 1.411)		1.054 (0.676, 1.643)		2.111 (1.182, 3.771)*
Family income per year						
<20,000 RMB		Reference		Reference		Reference
2–40000 RMB		1.129 (0.643, 1.983)		1.453 (0.753, 2.805)		0.679 (0.284, 1.623)
40000 RMB<		0.678 (0.389, 1.182)		1.111 (0.585, 2.110)		0.770 (0.324, 1.830)
Educational background						
Primary school		Reference		Reference		Reference
Junior high school		0.661 (0.223, 1.961)		6.350 (0.793, 50.814)		0.864 (0.160, 4.663)
High school		0.780 (0.243, 2.502)		3.884 (0.463, 32.611)		2.296 (0.362, 14.580)
College		0.581 (0.178, 1.897)		3.493 (0.408, 29.876)		5.157 (0.784, 33.907)
University and above		0.665 (0.163, 2.712)		2.054 (0.192, 22.007)		1.502 (0.175, 12.876)
Residential density	1.001 (1.000, 1.003)	1.001 (0.999, 1.002)	1.003 (1.001, 1.005)**	1.003 (1.000, 1.005)**	0.999 (0.997, 1.002)	0.997 (0.995, 1.000)
Land use mix diversity	1.117 (0.848, 1.470)	0.967 (0.708, 1.321)	0.986 (0.718, 1.354)	0.932 (0.654, 1.330)	1.124 (0.727, 1.737)	0.935 (0.556, 1.573)
Land use mix access	0.965 (0.629, 1.480)	1.042 (0.670, 1.621)	0.981 (0.596, 1.615)	1.000 (0.601, 1.663)	1.117 (0.586, 2.129)	1.356 (0.671, 2.740)
Street connectivity	0.791 (0.522, 1.198)	0.797 (0.516, 1.232)	0.599 (0.366, 0.980)*	0.598 (0.359, 0.995)*	1.207 (0.712, 2.046)	1.147 (0.652, 2.017)
Walking/cycling facilities	1.131 (0.825, 1.551)	1.008 (0.724, 1.402)	0.990 (0.688, 1.425)	0.951 (0.650, 1.391)	1.117 (0.720, 1.733)	0.892 (0.552, 1.441)
Aesthetics	1.156 (0.867, 1.540)	1.103 (0.819, 1.486)	0.986 (0.707, 1.375)	0.945 (0.669, 1.334)	1.145 (0.758, 1.730)	1.040 (0.668, 1.618)
Traffic safety	0.648 (0.416, 1.009)	0.714 (0.444, 1.146)	0.962 (0.576, 1.608)	0.980 (0.569, 1.687)	0.428 (0.226, 0.811)**	0.514 (0.257, 1.030)
Crime safety	0.987 (0.785, 1.240)	1.012 (0.799, 1.283)	1.044 (0.800, 1.362)	1.061 (0.806, 1.398)	1.397 (0.970, 2.013)	1.350 (0.912, 1.998)

^a^Model A only includes neighborhood environment. ^b^Model B also includes area, sex, family level, and educational background besides all the factors in Model A. *P < 0.05; **P < 0.01.
